# The incidence and risk factors of sessile serrated adenomas in left side colon cancer patients after curative surgery

**DOI:** 10.1097/MD.0000000000020799

**Published:** 2020-07-17

**Authors:** Myung Hee Kim, Hee Seok Moon, In Sun Kwon, Ju Seok Kim, Sun Hyung Kang, Jae Kyu Sung, Eaum Seok Lee, Seok Hyun Kim, Byung Seok Lee, Hyun Yong Jeong

**Affiliations:** a Division of Gastroenterology, Department of Internal Medicine, Chungnam National University School of Medicine; b Clinical Trials Center, Chungnam National University Hospital, Daejeon, South Korea.

**Keywords:** colorectal cancer, left side colectomy, serrated adenoma, sessile serrated adenoma

## Abstract

Sessile serrated adenomas (SSAs) are precursors of colorectal cancer (CRC). However, there are limited data on detection rates of this premalignant lesion during colonoscopy surveillance in patients with a history of left side colonic resection for cancer. We aimed to identify the incidence and risk factors of SSAs in post-left side colectomy patients.

We retrospectively reviewed the medical records of patients who had undergone left side colectomy for colon and rectal cancer between September 2009 and September 2016 and had at least 1 follow-up colonoscopy. Patient baseline characteristics, SSA diagnoses and characteristics, and colonoscopy information were collected.

In total, 539 patients were enrolled. At the first follow-up (mean duration 11.5 months), 98 SSAs were identified (22.2%). At the second follow-up (mean duration 25.8 months), 51 SSAs were identified in 212 patients (24.0%). Multivariate analysis showed that alcohol intake (hazard ratio [HR] 1.524; 95% confidence interval [CI] .963–2.411, *P* = .041), excellent bowel preparation (HR 2.081; 95% CI 1.214–3.567, *P* = .049), and use of a transparent cap (HR 1.702; 95% CI 1.060–2.735, *P* = .013) were associated with higher SSA incidence in the first surveillance colonoscopy, while body mass index (BMI) ≥ 25.0 (HR 1.602; 95% CI 1.060–2.836) was associated with a significantly increased risk of SSAs in the second surveillance.

Considering the endoscopic appearance of SSAs, adequate bowel preparation and use of transparent caps during postoperative surveillance colonoscopy can increase the diagnosis rate. Modification of alcohol intake and BMI may reduce the incidence of SSAs in left side colon cancer patients.

## 1 Introduction

**Figure 1:**
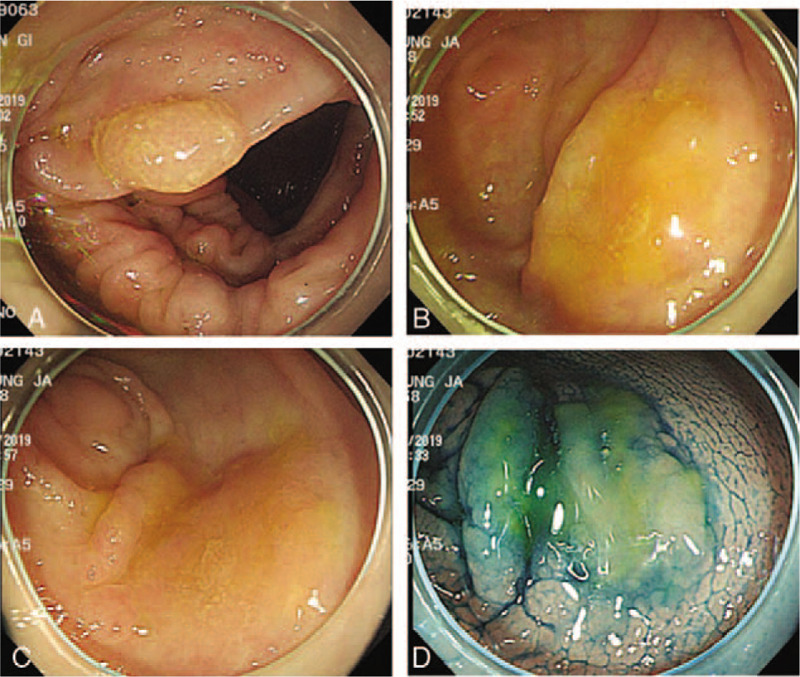
Morphologic characteristics of sessile serrated adenoma. A: Conventional endoscopy revealing a flat-elevated lesion with an 18-mm diameter covered with a mucus cap in the ascending colon. B: Endoscopic image revealing a pale, flat-elevated lesion (arrows) covered with mucus in the ascending colon. C: The target lesion is washed to sufficiently remove mucus, and a flat-elevated lesion with a 25-mm diameter is revealed. D: Chromoendoscopic image obtained after indigo carmine dye spraying.

Sessile serrated adenomas (SSAs) are important precursor lesions for colorectal cancer (CRC).^[1,2]^ Although SSAs are larger than hyperplastic polyps (HPs) on average, at least 1 study demonstrated that endoscopically, their flat morphology, indistinctive borders, and location in the proximal colon make detection during colonoscopy difficult (Fig. 1A–D).^[3]^ Furthermore, they are strongly associated with interval cancers. Thus, it is crucial to identify high-risk individuals and to carefully scrutinize them for SSAs during colonoscopies.

Surveillance colonoscopy represents a primary form of prevention and early detection of metachronous CRC.^[4]^ According to the US Multi-Society Task Force guidelines published in 2016, surveillance colonoscopy should be performed 1 year after CRC surgery. If there is no high-risk adenoma, a second surveillance colonoscopy can be performed 3 years later.^[5]^ This recommendation is based on large population-based studies, and the annual incidence of metachronous CRC is as high as 3%.^[6]^

Specifically, 1 study showed that the incidence of CRC was 2 times higher in patients with proximal primary CRC, whereas another study found a higher incidence of metachronous CRC in the proximal colon than in the distal colon.^[7]^ It is increasingly recognized that screening colonoscopy provides imperfect protection from CRC, especially those occurring in the proximal colon. Missed or rapidly growing lesions likely are important contributors to this problem.^[8]^ SSAs are more likely to be missing or incompletely removed than conventional adenomas.

Although there are many studies on the characteristics and incidence of SSAs, we can find no studies assessing the risk and incidence of SSAs based on post-operative surveillance colonoscopy. The incidence of adenoma is higher in patients with left side colon resection than in patients with right side colon resection, and SSAs are particularly difficult to detect in surveillance colonoscopy.^[9]^ Therefore, the aim of this study was to assess the risk of SSAs at the remnant site after left side colon resection.

## 2 Materials and methods

### 2.1 Study population

We retrospectively screened all consecutive patients who had undergone colonic surgery for colon and rectal cancer at Chungnam National University Hospital (CNUH) from September 2009 to September 2016. Inclusion criteria were as follows:

1. preoperative clearing colonoscopy was performed before colectomy, or if preoperative colonoscopy could not be performed because of obstruction, clearing colonoscopy was performed within 6 months after colectomy; and
2. at least the first surveillance colonoscopy was performed at CNUH at an interval consistent with the US Multi-Society Task Force guideline.

Patients diagnosed with inflammatory bowel disease, Lynch syndrome, or familial adenomatous polyposis, were excluded from the study. Patients with inadequate preparation (i.e., less than 90% of mucosa visualized) were also excluded. Endoscopic and demographic data were recorded at the time of the first and second surveillance colonoscopy, including sex, age, comorbidity (diabetes mellitus or hypertension), body mass index (BMI), alcohol and smoking status, TNM stage and differentiation of the index cancer, time interval between colectomy and the first and second surveillance colonoscopies, adequacy of bowel preparation and agent used, size, and number. The study protocol was approved by the institutional review board of CNUH (IRB number: 2019–02–025), and the requirement for informed consent was waived because of the retrospective design of the study.

### 2.2 Colonoscopy and pathological evaluations

The endoscopic and pathologic characteristics of polyps were reviewed and recorded. All colonoscopies were completed in the outpatient setting by an attending board-certified gastroenterologist or surgeon. In this study, more than 1000 colonoscopies were performed by both gastroenterologists and surgeons. The mean adenoma detection rates (ADRs) were all >25%, and a strict 6-minute CWT was maintained because of its importance as a colonoscopy quality indicator. All images were obtained with magnifying colonoscopies (CF-Q240ZI, and CF-H260AZI; Olympus, Tokyo, Japan) with up to 80-fold magnification, in combination with a standard video processor system (EVIS LUCERA system, EVIS EXERA system; Olympus Inc., Tokyo, Japan). Bowel preparation quality and preparation agents (Coolprep; Taejun Co., Seoul, South Korea) were recorded and analyzed. Bowel preparation quality was rated using the Aronchik scale as excellent, good, fair, or inadequate.

All polyps were characterized by size, shape, and location. They were removed using cold snare polypectomy and conventional endoscopic mucosal resection. One expert gastrointestinal pathologist classified polyps, based on histological criteria, as either an adenomatous (tubular, villous, and tubule villous) polyp or a serrated polyp (HP and SSA). The follow-up interval of surveillance colonoscopy was determined based on the clinical judgment of each colonoscopist. According to United States Multi-Society Task Force guidelines, high-grade dysplasias of tubular, and serrated adenomas, adenomas with at least 25% villous component, and adenomas larger than 10 mm were all considered advanced lesions.

### 2.3 Statistical analysis

Basic characteristics were summarized as mean and standard deviation for continuous variables and as frequency and percentage for categorical variables. The Student *t* test and Chi-Squared test were used for statistical assessment of continuous and categorical variables. Multivariate logistic regression analysis was performed to assess risk factors of SSA incidences in the first and second surveillance colonoscopy. All parameters with a *P* value of <.05 in the univariate analysis were included, whereas those with a *P* value of >.10 were removed by an automated stepwise procedure. SPSS software (version 22.0, IBM Corp., Armonk, NY, USA) was used for statistical analysis.

## 3 Results

### 3.1 Patient characteristics

**Figure 2:**
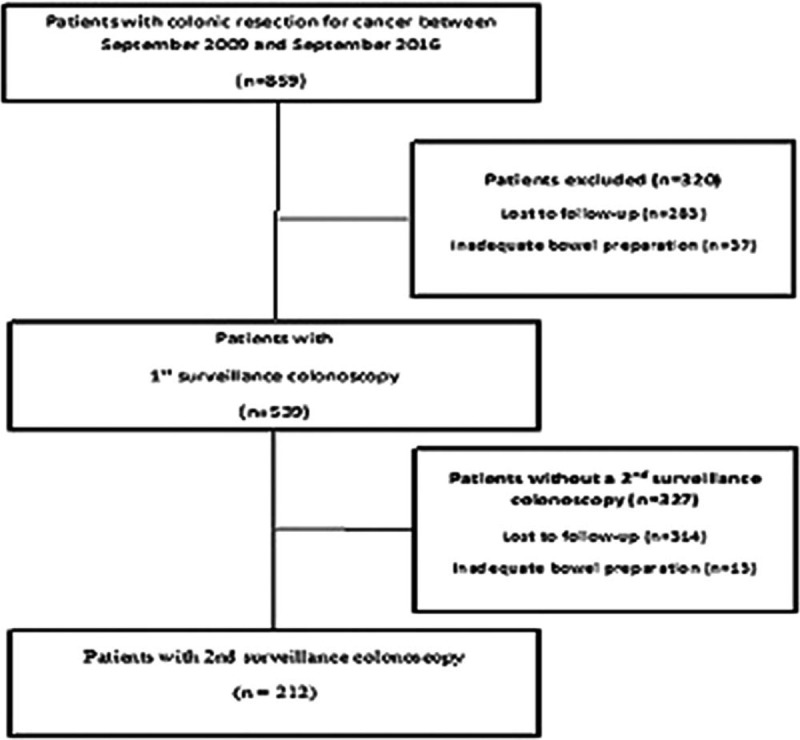
Flow diagram of enrolled patients.

Between September 2009 and September 2016, 859 patients underwent left colonic resection for CRC. After the exclusion of 320 patients because of follow-up loss or inadequate bowel preparation, 539 patients were included in the analysis (Fig. 2). The patients baseline characteristics are shown in Table 1. The mean age of patients with SSAs was 62.2 years, and 77% of patients were men. Colon cancer was the primary diagnosis in 445 patients (82.6%), and the remaining 94 patients (17.4%) had rectal cancer. Other characteristics of patients and tumor-related factors are presented in Table 1. Most patients underwent colonoscopy in the outpatient department; therefore, some data were missing in their medical records.

**Table 1:**
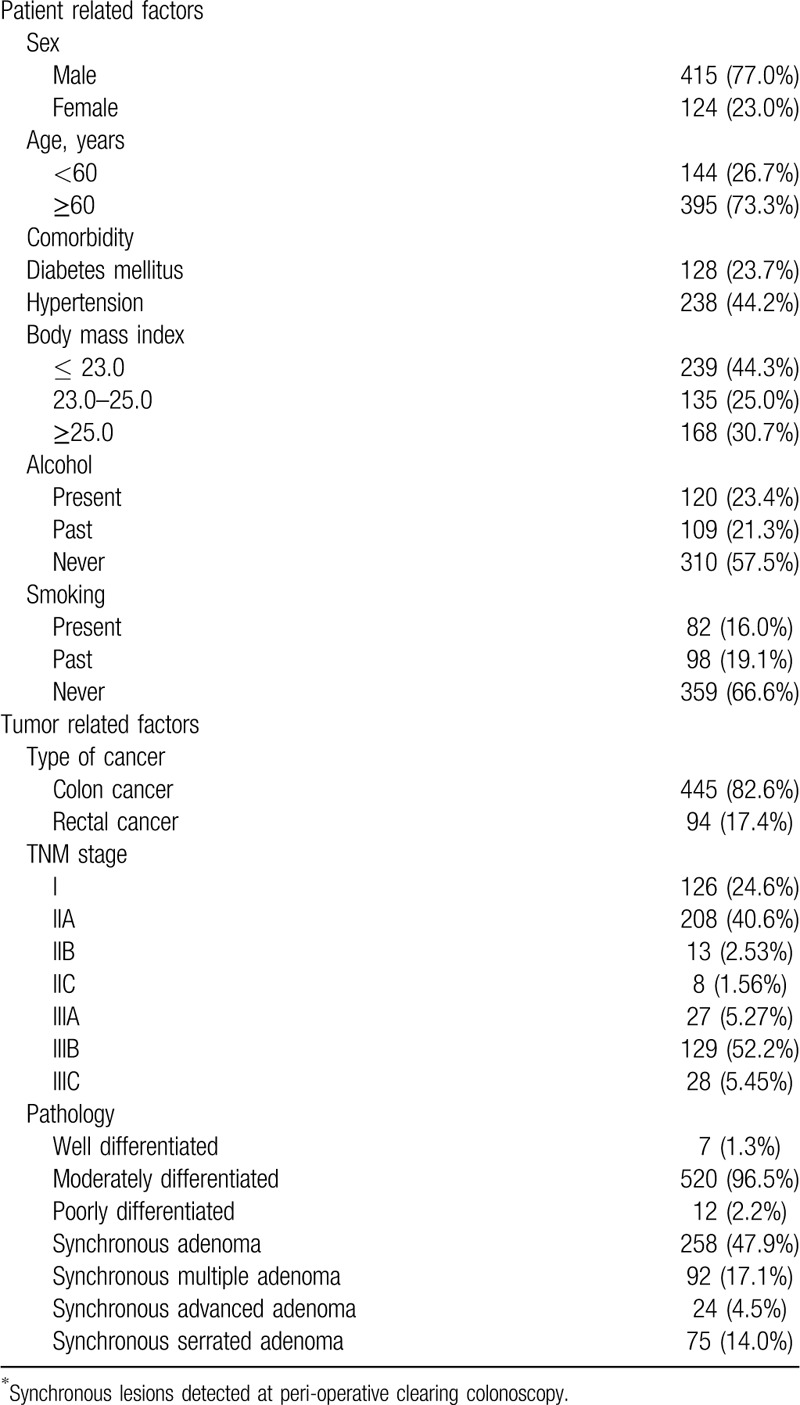
Baseline patient related factors (n = 539).

### 3.2 Incidence of SSA and follow-up colonoscopy period

The first mean follow-up duration was 11.5 months (95% confidence interval [CI], 11.1–11.6) and the second mean follow-up duration was 25.8 months (95% CI, 23.9–26.3) (Table 2). The total mean duration between the initial and final follow-up colonoscopies was 18.9 months, and the mean interval period between each colonoscopy was 12.8 months. At the first follow-up, 98 SSAs were identified in 539 patients (22.2%). At the second follow-up, 51 SSAs were identified in 212 patients (24.0%).

**Table 2:**
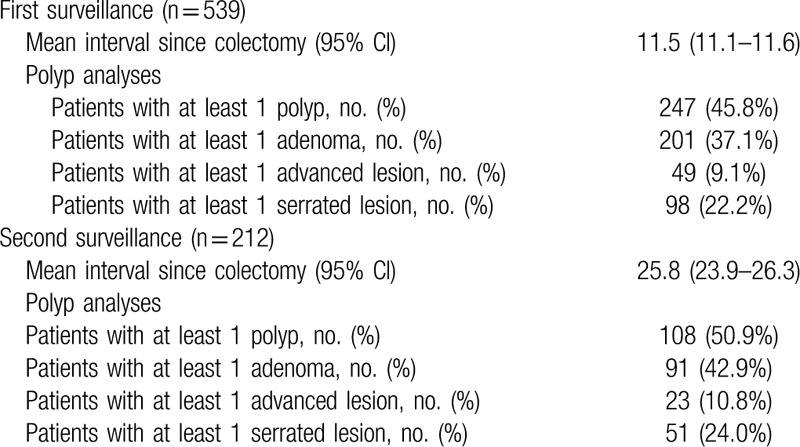
Surveillance colonoscopy intervals and polyp analyses stratified.

### 3.3 Analysis of SSA detection at first surveillance colonoscopy

SSA was found in 98/539 patients. Although not statistically significant, the incidence was higher in men (hazard ratio [HR] 1.662, 95% CI .933–2.963). The alcohol intake was the only significant lifestyle factor associated with greater SSA incidence (HR 1.940; 95% CI 1230–3.080, *P* = .035). Original tumors with TNM stage III were associated with greater SSA incidence (HR .514; 95% CI .280–.946, *P* = .032), as was use of a transparent cap (HR 1.789; 95% CI 1.150–2.783, *P* = .010). Multivariate analysis was performed using variables with a *P* value of ≤.1 in the univariate analysis. Multivariate analysis showed that alcohol intake (HR 1.524; 95% CI .963–2.411, *P* = .041), excellent bowel preparation (HR 2.081; 95% CI 1.214–3.567, *P* = .049) and use of a transparent cap (HR 1.702; 95% CI 1.060–2.735, *P* = .013) were associated with SSA incidence in the first surveillance colonoscopy (Table 3).

**Table 3:**
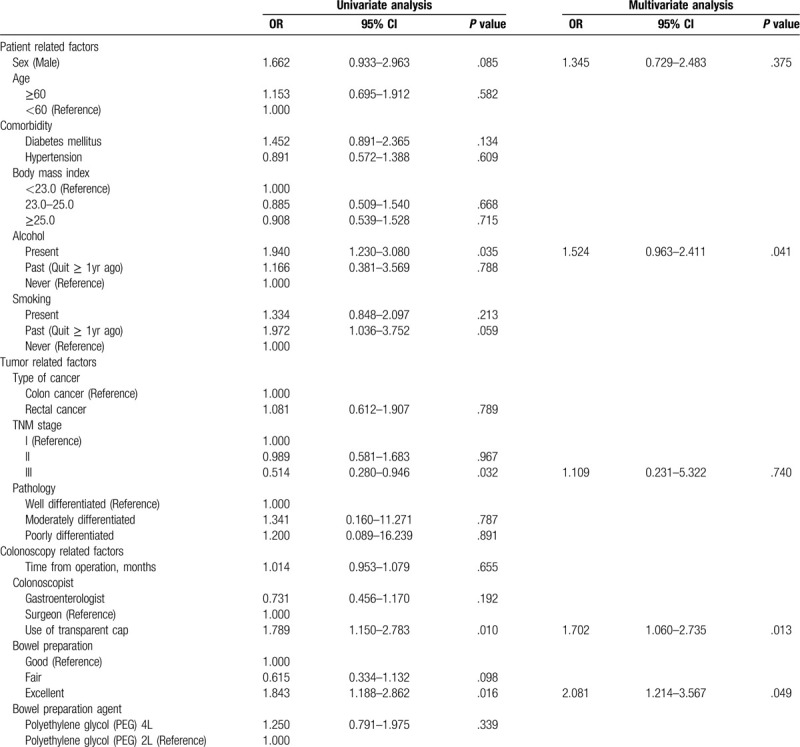
Analysis for the SSA detection at first surveillance colonoscopy (n = 98).

### 3.4 Analysis of SSA detection at second surveillance colonoscopy

A total of 212 patients underwent second surveillance colonoscopy, and 51 patients had SSAs. Age ≥ 60 years and BMI ≥ 25.0 kg/m^2^ were statistically significant in univariate analysis. The multivariate analysis revealed that BMI ≥ 25.0 kg/m^2^ (HR 1.602; 95% CI 1.060–2.836, *P* = .025) was associated with SSA recurrence after adjusting for other confounding factors. Age ≥ 60 years was not statistically significant in the multivariate analysis (Table 4).

**Table 4:**
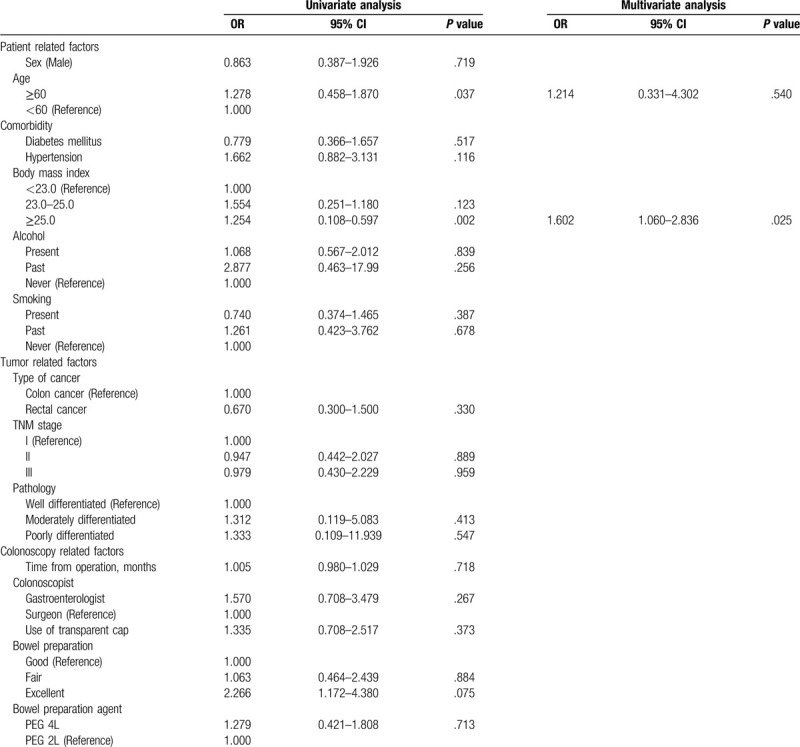
Analysis for the SSA detection at second surveillance colonoscopy (n = 51).

## 4 Discussion and conclusions

The most important goals of a CRC screening programs is the detection and removal of premalignant lesions. Post-operative surveillance colonoscopy is highly recommended for early treatment and is used to monitor recurrence at the anastomosis site and for early detection of metachronous adenomas and cancers.^[10]^ Recent data have determined that several distinct subtypes of serrated polyps are the precursors of a group of CRCs that exhibit hypermethylation and arise primarily in the proximal colon (i.e., the serrated neoplasia pathway); these CRCs are characterized by BRAF mutations and a CpG island methylator phenotype.^[11]^ It has been suggested that serrated polyps might be the precursors for approximately 15% to 20% of sporadic CRCs, particularly in the proximal colon.

Although most right CRC patients tend to have more microsatellite instability-high tumors, these tumors arise through the conventional adenoma pathway.^[12]^ Even though SSA occurred mainly in the right colon in this study, it is important to distinguish between the different genetic mechanisms of left CRC and SSA. However, some interval proximal cancers have been attributed to the serrated pathway, emphasizing the need to detect serrated polyps during surveillance endoscopic examinations.

Both SSA and traditional serrated adenomas have been recently recognized as the precursors of up to 20% of sporadic CRC through the serrated carcinoma pathway. Based on a recent report, SSAs comprise approximately 15% of all polyps seen on colonoscopy,^[13]^ and the ADR in specific screening cohorts is a validated quality indicator for colonoscopy performance.^[14]^ Secondary surveillance ADR after colorectal resection for colon cancer was significantly higher than first surveillance ADR colorectal resection for colon cancer.^[15]^ Moreover, Fuccio et al reported an ADR of 25.2% on second surveillance colonoscopy with a higher rate in those who had left hemicolectomy than right hemicolectomy.^[9]^ Furthermore, Yun et al also showed that left colon resection had a higher incidence of adenoma than right colon resection in the second surveillance colonoscopy.^[16]^ Because the incidence of adenoma in follow-up colonoscopy was significantly higher in patients with left colon cancer (arising from the descending and sigmoid colon, as well as the distal one-third of the transverse colon) than in patients with right colon cancer (arising from the ascending colon and proximal two-thirds of the transverse colon),^[17]^ the purpose of this study was to investigate the incidence and related factors of SSAs after left colon cancer operation.

Ninety eight SSAs (441 polyps) were detected in the first surveillance colonoscopies with a detection rate of 22.2%. The use of transparent caps and excellent bowel preparations were significantly associated with the detection of serrated adenomas. Adequate bowel preparation is associated with better detection of flat lesions, in general, and SSPs, in particular.^[18,19]^ This stands to reason, given their subtle endoscopic appearance and typical location in the right colon, which is more often affected by suboptimal bowel cleansing. The reason for this result is that the adenomas found in the first surveillance colonoscopy were synchronous and were already present at the time of the diagnosis, so they were associated with the characteristics of the serrated adenoma and their detection rate. The quality of colonoscopy has been strongly associated with the risk of post-colonoscopy CRC. Incomplete colonoscopy, rapid withdrawal time, and suboptimal adenoma detection rate have all been associated with a higher risk of post colonoscopy CRCs.^[19–21]^ One study found that a Boston Bowel Prep Scale (BBPS) score of 0 or 1 in any colon segment has a significantly higher rate of missed adenomas >5 mm.^[22]^ Therefore, inadequate bowel prep can lower ADR.^[23]^ We included a bowel prep grade from fair to excellent, as most colonoscopy reports did not include a BBPS score. Previous epidemiological data on serrated adenoma has consistently identified tobacco, alcohol, and obesity as risk factors for serrated polyps.^[24–26]^

In the second surveillance colonoscopy, analysis revealed that the higher the BMI, the higher the incidence of SSAs. A variety of inflammatory cytokines are produced in adipose tissue, some of which are carcinogenic. People with high BMI also have a high level of C-reactive protein, and a systematic review in 2008 found a direct link between C-reactive protein and CRC risk.^[27,28]^ This suggests that an increase in BMI increases the risk of developing CRC through increased inflammation. As serrated adenocarcinoma comprises 10% to 30% of all CRC, it is difficult to distinguish if this increased risk is mediated through one or more colorectal pathways. Within the abovementioned systematic review, some studies used alternative measurement methods for body fatness; therefore, further research is required using these alternative methods, particularly given suggestions that central adiposity may be of greatest importance for colorectal carcinogenesis. Another study showed that serum triglycerides are associated with the incidence of SSA. Because BMI tends to increase as blood triglyceride increases, weight loss accompanied by a reduction in the serum triglyceride levels may be effective for attenuating the development of SSAs and, therefore, preventing CRC development in obese subjects.^[29]^

Analyses for alcohol intake revealed a statistically significant increased risk of SSAs. After alcohol consumption, it undergoes metabolism to acetaldehyde via alcohol dehydrogenase and cytochrome P450 2E1 (CYP2E1).^[30]^ These enzymes are associated with a variety of cancers; however, in normal physiology, they play a role in the general detoxification of alcohol.^[31]^ Reduced alcohol intake is recommended to reduce serrated polyp and CRC risk.

In our study, we considered adenomas identified in the second surveillance colonoscopy as metachronous adenoma because factors affecting the incidence of serrated adenomas in the first surveillance colonoscopy were factors affecting detection; by contrast, in the second surveillance colonoscopy, incidence was related to lifestyle factors. Laiyemo et al reported that missed and recurrent adenomas are more likely to occur in the proximal colon. Missed adenomas were observed in the first surveillance colonoscopy conducted 1 year after surgery, and recurrent adenomas were observed in the second surveillance colonoscopy conducted 3 years later.^[32]^

Our study had several limitations that can be attributed to its retrospective design. First, because this was a single tertiary center study, the homogeneity of the population in this study could reduce the likelihood of generalizability. In addition, a large proportion of patients who underwent the first surveillance colonoscopy did not receive a second surveillance colonoscopy because of loss to follow-up. Therefore, the total number of patients enrolled was low. Second, because of the limitation of access to medical records, not all patients who underwent colon cancer surgery could be investigated because they did not undergo peri-operative colonoscopy at our center. Nevertheless, we attempted to perform colonoscopy within 6 months after colectomy in patients who did not have colonoscopy before surgery. Third, we only selected cases with serrated adenomas for the pathological reviews, and previously diagnosed HPs were excluded. A proportion of these lesions may have been misdiagnosed because of their serrated architecture, and therefore, the incidence of SSAs may have been underestimated.

Several measures were taken to reduce the impact of these limitations and strengthen our results. First, all colonoscopies were performed at our hospital by certified colonoscopists, thereby increasing the reliability of our results. Second, we only included cases with adequate bowel preparations, an important quality indicator. Third, we performed multivariate analysis of colonoscopy-related factors such as colonoscopist experience, usage of a transparent cap, bowel preparation quality, and bowel preparation agents. Investigation of these factors supports the theory that the SSA found on the first surveillance colonoscopy is a synchronous lesion. Fourth, the first and second surveillance colonoscopies were performed at 1 and 2 years after surgery, respectively. These are the appropriate surveillance intervals according to recent guidelines^[5]^; on average, the second surveillance colonoscopy was performed 1 year earlier than guidelines specify. Finally, our study was limited to the incidence of SSAs and risk factors in patients who underwent left colon resection. Although this may be a disadvantage, SSA is most likely to occur in the proximal colon, and it is easy to overlook these lesions because of the nature of the endoscopic morphology.

In conclusion, using data analytics, we found that SSAs are more likely to be present in individuals who are obese and are users of alcohol. By connecting these risk factors to specific polyp subgroups, our results may help to identify high-risk groups that require CRC surveillance. Individuals who have these risk factors should be carefully scrutinized for the presence of SSAs during surveillance colonoscopies. Moreover, in relation to modifiable risk factors, reductions of weight and alcohol intake should be recommended.

## Acknowledgments

The authors thank Editage (www.editage.com) for English language editing.

## Author contributions

Hee Seok Moon designed the study and wrote the initial draft of the manuscript. Myung Hee Kim contributed to the analysis and interpretation of data and assisted in the preparation of the manuscript. All other authors contributed to data collection and interpretation and critically reviewed the manuscript. All authors approved the final version of the manuscript, and agree to be accountable for all aspects of the work in ensuring that questions related to the accuracy or integrity of any part of the work are appropriately investigated and resolved.

## Author contributions

**Conceptualization:** Hee Seok Moon.

**Data curation:** Myung Hee Kim, Eaum Seok Lee.

**Formal analysis:** Myung Hee Kim,

**Investigation:** Ju Seok Kim, Sun Hyung Kang, Eaum Seok Lee, Jae Kyu Sung.

**Methodology:** Hee Seok Moon, In Sun Kwon.

**Supervision:** In Sun Kwon, Seok Hyun Kim, Byung Seok Lee, Hyun Yong Jeong

**Writing – original draft:** Myung Hee Kim

**Writing – review & editing:** Hee Seok Moon.
